# Homologous recombination deficiency (HRD) testing in ovarian cancer clinical practice: a review of the literature

**DOI:** 10.1186/s40661-017-0039-8

**Published:** 2017-02-22

**Authors:** Melissa K. Frey, Bhavana Pothuri

**Affiliations:** 1 Division of Gynecologic Oncology, Weill Cornell Medicine, 525 East 68th Street, Suite J-130, New York, NY 10065 USA; 2 Division of Gynecologic Oncology, New York University Langone Medical Center, 240 E. 38th St, 19th floor, New York, NY 10016 USA

**Keywords:** Ovarian cancer, Genetics, Tumor testing

## Abstract

Until recently our knowledge of a genetic contribution to ovarian cancer focused almost exclusively on mutations in the *BRCA1/2* genes. However, through germline and tumor sequencing an understanding of the larger phenomenon of homologous recombination deficiency (HRD) has emerged. HRD impairs normal DNA damage repair which results in loss or duplication of chromosomal regions, termed genomic loss of heterozygosity (LOH). The list of inherited mutations associated with ovarian cancer continues to grow with the literature currently suggesting that up to one in four cases will have germline mutations, the majority of which result in HRD. Furthermore, an additional 5–7% of ovarian cancer cases will have somatic HRD. In the near future, patients with germline or somatic HRD will likely be candidates for a growing list of targeted therapies in addition to poly (ADP-ribose) polymerase (PARP) inhibitors, and, as a result, establishing an infrastructure for widespread HRD testing is imperative. The objective of this review article is to focus on the current germline and somatic contributors to ovarian cancer and the state of both germline and somatic HRD testing. For now, germline and somatic tumor testing provide important and non-overlapping clinical information. We will explore a proposed testing strategy using somatic tumor testing as an initial triage whereby those patients found with somatic testing to have HRD gene mutations are referred to genetics to determine if the mutation is germline. This strategy allows for rapid access to genomic information that can guide targeted treatment decisions and reduce the burden on genetic counselors, an often limited resource, who will only see patients with a positive somatic triage test.

## Background

Ovarian cancer is the fifth leading cause of death in women in the United States with an estimated 22,280 new cases and 14,240 deaths in 2016 [1]. The majority of women with epithelial ovarian cancer (EOC) are diagnosed with advanced disease. Standard therapy includes surgical debulking and platinum and taxane-based chemotherapy, resulting in complete clinical remission in up to 75% of patients, however only 30% of patients will be cured. Once recurrent, ovarian cancer generally does not exhibit the same level of chemo-sensitivity, highlighting the need for rational therapies directed toward specific molecular targets [2, 3]. The advent of next-generation sequencing (NGS) has allowed for the systematic investigation of the genomic and molecular alterations in EOC which can identify patients as candidates for individualized targeted therapy. EOC tumors with deficient homologous recombination (HR) repair represent such a group and have demonstrated sensitivity to poly(ADP-ribose)polymerase (PARP) inhibitors [4–6]. The majority of homologous recombination deficient (HRD) tumors will occur in patients with germline *BRCA1* and *BRCA2* mutations. However, there also are patients with germline mutations in other HR pathway genes and patients who do not carry an inherited germline mutation but have tumors with sporadic HRD mutations. Data from the Cancer Genome Atlas (TCGA) demonstrates that approximately fifty percent of high grade serous ovarian cancers have aberrations in HR repair [7]. Patients and physicians now have access to NGS analysis of both germline samples and somatic tumor tissue. The objective of this review article is to focus on the current germline and somatic contributors to ovarian cancer and the state of germline and somatic HRD testing.

### Homologous recombination (HR)

DNA damage is a constantly occurring phenomenon that necessitates a complex network of molecular repair pathways in order to maintain genomic integrity and prevent cell death. HR is an important pathway that allows repair of double-stranded DNA breaks. HR operates during the S and G2 phases of the cell cycle and relies on many proteins including *BRCA1* and *BRCA2*, proteins of the MNR complex (*MRE11/RAD50/NBS1*), *CtIP, MRE11, RAD51, ATM, H2AX, PALB2, RPA, RAD52* and proteins of the Fanconi anemia pathway [8, 9]. When cells have non-functioning HR, for example due to *BRCA1* or *BRCA2* deficiency, they rely on other repair pathways like non-homologous end-joining (NHEJ), which is less precise and more error-prone [10]. NHEJ results in the accumulation of additional mutations and chromosomal instability, increasing the risk that a cell undergoes malignant transformation [10, 11].

Until recently, hereditary EOC was thought to be caused almost exclusively by mutations in *BRCA1* and *BRCA2*, with a small contribution from mutations in the DNA mismatch repair (MMR) genes [12]. TCGA, however, has shown that about half of high grade serous ovarian cancers, the most common histologic subtype, have aberrations in HR repair. Further investigation of the HR pathway highlights multiple other protein co-factors that are necessary for successful HR repair including *RAD51C*, *RAD51D*, *BRIP1*, *PALB2*, *BARD1* and the MMR genes [12, 13]. This group of genes is collectively referred to as the HRD genes [14].

### Germline mutations in EOC

When considering the genetic contribution to carcinogenesis, an important distinction is whether a mutation is germline or somatic. Germline implies that the mutation was inherited and is therefore present in all of the individuals’ cells. Somatic mutations are those mutations that are acquired and therefore occur exclusively in the tumor cells. *BRCA1* and *BRCA2* are the most well-known ovarian cancer susceptibility genes, with germline genetic testing available since the 1990s [15]. *BRCA* mutation-associated ovarian cancers have multiple distinct clinical features including earlier age at diagnosis, visceral distribution of disease, improved survival, enhanced sensitivity to platinum-based chemotherapies and sensitivity to PARP inhibitors [16–18]. The prevalence of *BRCA1/2* germline mutations in patients with epithelial ovarian cancer is estimated to be about 11–15% [19–21].

In June 2013 the Supreme Court ruled unanimously against Myriad Genetics, invalidating the exclusive license rights to *BRCA1* and *BRCA2* testing in the United States. Following this decision, many other clinical laboratories began offering *BRCA1* and *BRCA2* testing, both as single gene tests and in the form of comprehensive genetic panels [22]. Although used for many hereditary cancer syndromes, multigene panels have been particularly interesting in ovarian cancer. Recent literature suggests that up to 24% of ovarian cancers are associated with germline mutations, and of these, 29% have mutations in genes other than *BRCA1* or *BRCA2* [12, 13, 23, 24] (Table 1).

**Table 1:** Genes associated with hereditary ovarian cancer

Hereditary breast and ovarian cancer syndrome
BRCA1
BRCA2
Fanconi anemia pathway
RAD51C
RAD51D
RAD50
BRIP1
BARD1
CHEK2
MRE11A
NBN
PALB2
Mismatch repair
MLH1
MSH2
MSH6
PMS2
Other
TP53

### Somatic mutations in EOC

Several publications have reported the presence of somatic *BRCA1/2* mutations in ovarian cancer, highlighting that both germline and somatic mutations in HRD genes can result in ovarian cancer. TCGA sequenced 316 high grade serous EOCs and matched germline patient DNA. Germline *BRCA1* mutations were discovered in 9% of patients and *BRCA2* mutations in 8%. Evaluation of tumor tissue found additional somatic mutations in *BRCA1/2* (3%), *EMSY* (8%), *PTEN* (7%), *RAD51C* (3%), *ATM/ATR* (2%) and Fanconi anemia genes (5%) [7]. Hennessy et al. [25] performed *BRCA1* and *BRCA2* sequencing of 235 unselected ovarian cancer tumors and found mutations in 19% of cases (31 mutations in *BRCA1* and 13 in *BRCA2*). Germline DNA specimens were available for 28 of the patients harboring a *BRCA1* or *BRCA2* tumor mutation. Eleven (39.3%) of the mutations present in the EOC tumor were found to be somatic and 17 germline (60.7%) [25]. When evaluating 367 ovarian carcinomas, Pennington et al.[26] discovered that 24% patients carried a germline mutations in HRD genes and 9% of patients had a somatic tumor mutation. Finally, Cunningham et al. [27] evaluated 279 EOC patients and found that 3.5% of patients had somatic mutations in *BRCA1* (2.1%) and *BRCA2* (1.4%). (Table 2). The variable rates of somatic mutations reported in the literature likely reflect differences in patient selection (e.g., histologic subtype) and scope of testing (e.g., number of genes included on the NGS panel). Currently, the true prevalence of somatic mutations remains unknown, however it has been estimated as somewhere between 5 and 7% of cases. This implies that for every 4–5 ovarian cancer patients with a germline BRCA mutation there will be one patient with a somatic mutation [8].

**Table 2:** Germline and somatic HRD mutations in ovarian cancer

Study	Included histologic subtypes	Findings
TCGA [7]	High grade serous ovarian cancer (316)	Germline mutations (*N* = 316) BRCA1 – 9% BRCA2 – 8%
		Somatic mutations (*N* = 216) BRCA1/2–3% EMSY – 8% PTEN – 7% RAD51C – 3% ATM/ATR – 2% Fanconi anemia genes – 5%
Hennessy et al. [25]	Serous (186) Nonserous (13) Mixed (13) Unknown (22)	Tumor sequencing (*N* = 235) BRCA1 – 13% BRCA2 – 5.5%
		Germline testing from patients with tumors harboring BRCA1/2 mutations (*N* = 28) BRCA1/2 germline mutation – 61% BRCA1/2 somatic mutation – 39%
Pennington et al. [26]	High grade serous (249) Low grade serous (9) Poorly-differntiated NOS (48) Clear cell (19) High-grade endometrioid (20) Low-grade endometrioid (6) Carcinosarcoma (12) Other (4)	Germline mutations (*N* = 367) HRD genes – 24% (BRCA1 – 56%, BRCA2 – 19%, BARD1 – 2%, 4, BRIP1 – 4.5%, CHEK1 – 1%, CHEK2 – 3%, FAM175A – 2%, NBN – 1%, PALB2 – 2%, RAD51C – 3%, RAD51D – 4.5%)
		Somatic Mutations (*N* = 367) HRD genes – 9% (BRCA1 – 54%, BRCA2 – 17%, ATM – 9%, BRIP1 – 6%, CHEK2 – 9%, MRE11A – 3%, RAD51C – 3%)
Cunningham et al. [27]	High grade serous (735) High grade endometrioid (73) Low grade endometrioid (67) Clear cell (69) Low-grade serous (34) Mucinous (29) Other/Unknown (56)	Germline mutations (*N* = 899) BRCA1 – 3.5% BRCA2 – 3% RAD51C – 3%
		Somatic Mutations (*N* = 279) BRCA1 – 2% BRCA2 – 1.4%

### Genetic evaluation

#### Germline testing

Genetic testing for germline mutations is recommended for all women with non-mucinous epithelial ovarian cancer by multiple professional societies including the American College of Medical Genetics and Genomics (ACMG) [28], the American Society of Clinical Oncology (ASCO) [29], the National Cancer Comprehensive Network (NCCN) [30], the National Society of Genetic Counselors (NSGC) [28] and the Society of Gynecologic Oncology (SGO) [31]. Multigene panels can assess a virtually unlimited number of genes in a method that is both time- and cost-effective when compared to the prior standard of single gene Sanger sequencing. The availability of NGS technology combined with the Supreme Court ruling invalidating exclusive gene patent rights has resulted in rapid uptake of multigene panels [32]. The scope of available testing ranges from screening for founder mutations, to sequencing *BRCA1* and *BRCA2*, to full panel testing of multiple cancer-associated genes.

Currently, the guidelines that recommend germline testing do not make specific recommendations regarding which of the many available platforms to utilize [14]. Cancer genetic counselors play an integral role both in helping patients choose the specific method of testing and analyzing the results, which are becoming increasingly complex. While adding additional genes to panels increases the chance of finding deleterious mutations it also increases the likelihood of finding variants of uncertain significance (VUS) and non-actionable mutations defined as mutations for which the clinical relevance is unclear and clinical management guidelines do not exist. Finding VUS and non-actionable mutations does not improve patient outcomes but can result in patient and physician anxiety and unindicated and inappropriate interventions [33]. Pre- and post-test cancer genetic counseling has been recognized to benefit the individual tested and relatives, associated with improved adherence to cancer risk management, better informed surgical decision making, increased cancer genetics knowledge, improved patient satisfaction and cost savings [34–46].

Discovering a germline mutation in a patient with EOC has multiple significant clinical implications. As previously stated, *BRCA1/2*-associated ovarian cancer has multiple distinct features affecting age at diagnosis, disease distribution, chemosensitivity and survival. Patients with known mutations have access to targeted therapy, for example PARP inhibitors for *BRCA1/2* mutation carriers. In December 2014, the U.S. Food and Drug Administration (FDA) granted olaparib accelerated approval for monotherapy in patients with germline *BRCA1/2* mutations and recurrent ovarian cancer with three or more prior lines of chemotherapy, making olaparib the first of the PARP inhibitors to receive FDA approval. On December 19, 2016 the FDA granted accelerated approval to rucaparib in patients with recurrent ovarian cancer with two or more prior lines of chemotherapy and either germline *BRCA1/*2 mutations or somatic *BRCA1/*2 mutations in the tumor detected by a next-generation sequencing-based companion diagnostic test. The European Commission granted marketing authorization for the PARP inhibitor olaparib as monotherapy in the maintenance treatment of adult patients with platinum-sensitive, relapsed *BRCA*-mutated (germline and/or somatic) high-grade serous epithelial ovarian, fallopian tube, or primary peritoneal cancer who are in complete response or partial response following platinum-based chemotherapy [47]. The recently published phase III data of Mirza et al. support the use of Niraparib in the maintenance setting for patients with platinum sensitive recurrent ovarian cancer. Patients with germline HRD mutations derived the greatest clinical benefit from this treatment [48].

In addition to treatment eligibility, many mutations are predictive of other cancers in addition to ovarian cancer, offering patients the opportunity for heightened awareness and screening. Finally, identifying a germline mutation can allow family members to undergo genetic counseling and testing, termed “cascade” testing. Informed relatives have the opportunity to pursue intensive cancer surveillance and risk-reducing options. Data from individuals with Lynch syndrome demonstrate that awareness of a mutation results in cancer prevention or early diagnosis of cancer, which can improve morbidity and prevent mortality [49–51].

#### Tumor profiling

In addition to germline testing, multiple companies now offer tumor genetic profiling for somatic mutations. There are methods using NGS similar to that done for germline DNA. There are also independent DNA-based measures of genomic instability reflecting underlying tumor HRD on the basis of loss of heterozygosity (LOH), telomeric allelic imbalance (TAI) and large-scale state transitions (LST). Each individual metric and the combination is significantly associated with *BRCA1/2* status and identifying HRD tumors [52–54]. Somatic mutation testing is more laborious and less reproducible than germline testing for several reasons. First, unlike germline testing, somatic testing utilizes a heterogeneous collection of cells. Different tumor types and specimens will have varying quantities of normal cells and neoplastic cells in a sample. Multiple methods of DNA evaluation exist for tumor profiling. Obtaining high-quality tumor DNA extraction relies on an adequately sized specimen that has been well preserved. In contrast to germline testing which looks at DNA extracted from healthy cells to determine if a mutation was inherited, somatic testing evaluates the genetic composition of tumor cells. Once adequate high-quality DNA has been obtained the data analysis is also challenging. There is currently no standard method of interpreting results. Laboratories rely on databases such as COSMIC or in silico predictive software to attempt to predict pathogenicity of findings but neither is exhaustive [8]. Finally there is the issue of the stability of a somatic mutation. Data suggest that there is intra-tumor genetic heterogeneity resulting from clonal evolution and the emergence of subclonal tumor populations in high grade EOC, causing spatial and temporal heterogeneity [55]. While data from the ARIEL-2 trial has begun to address this issue, demonstrating a change in biomarker status over time, the phenomenon of tumor heterogeneity is not fully understood [56].

Early evidence suggests that a somatic *BRCA1/2* mutation is a predictive biomarker of PARP inhibitor activity however the number of patients with somatic mutations that have been analyzed is low. In a phase II study of olaparib, Gelmon et al. found that 24% of *BRCA*-negative patients with high grade serous or undifferentiated ovarian cancer achieved a radiological objective response [57]. In the recent publication by Mirza et al. [48] niraparib maintenance therapy had activity in all patients with platinum sensitive recurrent ovarian cancer, however there was improved progression free survival in patients with germline *BRCA1/2* mutations and without germline *BRCA1/*2 mutations but with HRD. Swisher et al. [59] found that for patients with platinum sensitive recurrent ovarian cancer, rucaparib had activity in patients with germline *BRCA1/*2 mutations and patients who were *BRCA1/*2 wild-type but were found to be LOH high. LOH status was determined by next-generation sequencing using a cutoff of 14% or more of genomic LOH in pretreatment biopsy specimens to be considered LOH high. Studies that are currently ongoing or recently completed, and future planned studies will likely clarify this question, including SOLO-2 (NCT01874353) and ARIEL-3 (NCT01968213) [5, 60]. See Table 3 for a review of phase II/III trials of PARP inhibitors in ovarian cancer.

**Table 3:** Phase II/III studies of PARP inhibitors in ovarian cancer

Study	Patient population	BRCA status of patient population	Treatment arms	Total accrual	Primary endpoint	Results		
						Objective Response Rate (ORR)	Progression free survival (PFS)	Pertinent Findings
Audeh MW et al. Lancet. 2010 [4].	Recurrent epithelial ovarian, primary peritoneal, or fallopian tube carcinoma	BRCA1/2 positive	Cohort 1: Olaparib 400 mg BID Cohort 2: Olaparib 100 mg BID	57	ORR	Cohort 1: 33% Cohort 2: 13%	Cohort 1: 5.8 months Cohort 2: 1.9 months	Positive proof of concept of utility of PARP inhibitors from phase I data. Superior efficacy of 400 mg BID dosing.
Kaye SB et al. J Clin Oncol. 2012 [73].	Platinum resistent recurrent epithelial ovarian, primary peritoneal, or fallopian tube carcinoma	BRCA1/2 positive	Arm 1: Olaparib 200 mg BID Arm 2: Olaparib 400 mg BID Arm 3: PLD 50 mg/m2	97	PFS	Arm 1: 25% Arm 2: 31% Arm 3: 18%	Arm 1: 6.5 months Arm 2: 8.8 months Arm 3: 7.1 months	No significant difference in outcomes between 2 doses of olaparib and PLD.
Gelmon KA et al. Lancet Oncol. 2011 [57].	Advanced metastatic or recurrent ovarian, primary peritoneal or fallopian tube cancer (high-grade serous and/or undifferentiated) or breast cancer	BRCA1/2 positive AND BRCA1/2 negative	Olaparib 400 mg BID	91 (65 with gynecologic cancer)	ORR	BRCA1/2 positive: 41% BRCA1/2 negative: 24% BRCA1/2 positive + platinum sensitive: 60% BRCA1/2 negative + platinum sensitive: 50% BRCA1/2 positive + platinum resistant: 33% BRCA1/2 negative + platinum resistant: 4%	BRCA1/2 positive: 221 days BRCA1/2 negative: 192 days	Olaparib has activity in BRCA1/2 positive and negative populations.
Ledermann J et al. Lancet Oncol. 2014 [5].	Platinum sensitive recurrent high grade serous epithelial ovarian, primary peritoneal, or fallopian tube carcinoma	BRCA1/2 positive AND BRCA1/2 negative	(Maintenance therapy following platinum-based chemotherapy) Arm 1: Olaparib 400 mg BID Arm 2: Placebo	265	PFS		Arm 1: 8.4 months Arm 2: 4.8 months Olaparib + BRCA1/2 positive: 11.2 months Olaparib + BRCA1/2 negative: 5.6 months Placebo + BRCA1/2 positive: 4.3 months Placebo + BRCA1/2 negative: 5.5 months	Olaparib maintenance associated with improved PFS. No diffrence in OS.
Oza AM et al. Lancet Oncol. 2015 [74].	Platinum sensitive recurrent serous ovarian cancer	BRCA1/2 positive AND BRCA1/2 negative	Arm 1: Olaparib 200 mg BID + Paclitaxel 175 mg/m2 + Carboplatin AUC 4 × 6 cycles followed by olaparib 400 mg BID maintenance Arm 2: Paclitaxel 175 mg/m2 + Carboplatin AUC 4 × 6 cycles	162	PFS	Arm 1: 64% Arm 2: 58%	Arm 1: 12.2 months Arm 2: 9.6 months	Olaparib associated with improved PFS.
Coleman RL et al. Gynecol Oncol. 2015 [75].	Recurrent or persistent ovarian, primary peritoneal or fallopian tube cancer	BRCA1/2 positive	Veliparib 400 mg BID	52	ORR	Total population – 26% Platinum resistant – 20% Platinum sensitive – 35% BRCA1 – 26% BRCA2 – 27%	8.11 months	Veliparib has single agent acitivity in platinum resistent disease.
Kaufman B et al. J Clin Oncol. 2015 [76].	Platinum resistent recurrent ovarian, primary peritoneal or fallopian tube cancer	BRCA1/2 positive	Olaparib 400 mg BID	193	ORR	31%	225 days	Olaparib has single agent acitivity in BRCA1/2 positive platinum resistent disease.
Kummar S et al. Clin Cancer Res 2015 [58].	Recurrent ovarian cancer or recurrent primary peritoneal, fallopian tube or high-grade serous ovarian cancers	BRCA1/2 positive AND BRCA1/2 negative	Arm 1: Cyclophosphamide 50 mg daily Arm 2: Cyclophosphamide 50 mg daily + Veliparib 60 mg daily	75	ORR	Arm 1: (*n* = 38) 1 complete response, 6 partial responses Arm 2: (*n* = 37) 1 complete response, 3 partial responses	Arm 1: 2.3 months Arm 2: 2.1 months	The addition of veliparib to cyclophosphamide did not improve the response rate or the median PFS.
Mirza MR et al. N Engl J Med 2016 [48].	Platinum sensitive recurrent ovarian cancer or recurrent primary peritoneal, fallopian tube or high-grade serous ovarian cancers	BRCA1/2 positive AND BRCA1/2 negative	Niraparib 300 mg daily vs. placebo daily	553	PFS		gBRCA cohort - Niraparib: 21.0 months - Placebo: 5.5 months non-gBRCA with HRD cohort - Niraparib: 12.9 months - Placebo: 3.8 months non-gBRCA cohort - Niraparib: 9.3 months - Placebo: 3.9 months	Niraparib maintenance therapy has activity for platinum-sensitive recurrent ovaruan cancer regardles of the presence or absense of gBRCA mutations or HRD status.
Swisher EM, et al. Lancet Oncol 2017 [59].	Platinum sensitive recurrent ovarian cancer or recurrent primary peritoneal, fallopian tube or high-grade ovarian cancers	BRCA1/2 positive AND BRCA1/2 negative	Rucaparib 600 mg BID	206	PFS	BRCA 1/2 positive – 80% BRCA1/2 wild-type and LOH high – 29% BRCA1/2 wild-type and LOH low – 10%	BRCA 1/2 positive: 12.8 months BRCA1/2 wild-type and LOH high: 5.7 months BRCA1/2 wild-type and LOH low: 5.2 months	Rucaparib acitivity in BRCA1/2 mutant and BRCA wild-type LOH high platinum sensitive recurrent disease.

*gBRCA* Germline BRCA mutation, *non-gBRCA* Non-germline BRCA mutation, *LOH* Loss of heterozygosity

Somatic tumor testing has two important implications: 1) identifying patients for targeted therapies like PARP inihibitors, 2) identifying patients who should be referred for genetic counseling and offered genetic testing. Mutations that are purely somatic and not found in the germline DNA are not inherited, cannot be passed to offspring, and therefore do not warrant cascade genetic counseling and testing. If a patient is found to have a mutation on tumor profiling, the only way to definitively classify it as a germline versus somatic mutation is through germline testing. As described previously, Hennessy et al. [25] found that among 28 tumors with *BRCA1/2* mutations, 61% were the result of germline inherited mutations. This presents an important ethical consideration regarding patient counseling and informed consent. Thorough genetic counseling and informed consent are required prior to germline genetic testing however this is not the case with tumor profiling. This is important given that almost two-thirds of patients with a mutation found on tumor profiling will have a germline mutation which carries significant clinical implications for the patient and her family members.

#### Tumor profiling vs. germline testing?

It has become clear that both somatic and germline mutation testing provide important information for patients with EOC. The question is whether or not both are necessary and if so what is the ideal chronology of testing. Table 4 outlines the advantages of each testing method. Germline testing is more well established with more robust data linking results to valuable prognostic and predictive information. Germline testing can also identify other cancers for which a patient is at risk and can initiate cascade testing of family members. Somatic testing will capture a larger number of patients with HRD who can potentially receive targeted therapy and does not require access to a genetic counselor, which is limited in many clinical settings.

**Table 4:** Advantages of germline versus somatic tumor testing of HRD genes

Advantages of germline testing
1. Germline testing is a more well established technique.
DNA extraction is easier.
The results are highly accurate and reproducible.
2. Germline mutations have prognostic and predictive value.
There is robust data supporting the prognostic and predictive value of germline *BRCA1/2* mutations.
Such data is limited for somatic mutations.
3. Germline mutations can offer knowledge of risk for other associated cancers.
As germline mutations often increase the risk for multiple cancers, awareness of germline mutations allows patients to pursue risk-reducing interventions for other cancers.
4. Germline mutation identification is clinically relevant for family members and allows for cascade testing.
Advantages of somatic testing
1. Somatic testing with NGS will identify a larger number of patients with HRD who can be therapeutically targeted.
Including patients with somatic (and not germline) mutations who would be missed with germline testing alone.
2. Somatic testing can help patients understand the magnitude of clinical benefit from targeted therapy in the context of risks and side effects of particular therapeutic agents.
3. Somatic testing does not require genetic counseling which is often a limited resource.
4. Somatic testing can serve as a triage for germline testing.
Patients found to have a somatic mutation can then be referred to clinical genetics for germline testing, allowing for better utilization of genetic counselors and geneticists.

This issue of genetic counseling as a limited resource is an important one. The exponential growth in diagnostic and treatment options that utilize genetic and genomic sequencing information coupled with public coverage of celebrity *BRCA* mutation status has led to an increasing demand for genetic counselors [61, 62]. A study of 3765 patients in the U.S. with epithelial ovarian cancer found that only 50% of patients meeting substantial-risk criteria for *BRCA1/2* mutations were referred to genetics [63]. Another study of 416 patients in Ontario with epithelial ovarian cancer found that only 19% of patients had undergone clinical genetic testing for *BRCA1/2* [64]. There currently are not enough genetic counselors and geneticists to meet this demand and alternative service delivery models are being evaluating including telephone counseling, videoconferencing, group counseling and direct-to-consumer testing [65]. Video-assisted genetic counseling has been found to significantly increase the percentage of patients undergoing testing compared to traditional referral to genetic counseling (31% vs. 55%) however this method has not yet been tested in a prospective manor [66].

The recent approval of rucaparib for BRCA positive (germline or somatic) patients with recurrent ovarian cancer who have had two or more lines of therapy would argue for tumor testing to screen for therapeutic options and as a triage for germline testing. However, the success of niraparib in significantly extending the duration of progression-free survival in all platinum-sensitive recurrent ovarian cancer patienrs, (those with and without *BRCA1/2* mutations) indicates a great expansion of the population who can benefit from PARP inhibitors. Interestingly Mirza et al. even found efficacy in the non-germline BRCA mutation and HRD-negative group (HR 0.58, *P* = 0.02). In an exploratory analysis of ARIEL2 Part 1, Swisher et al. [59] found that among BRCA wild-type tumors, genomic LOH was a more sensitive predictor of response than was mutation of other HR genes and gene methylation. In the near future PARP inhibitors may be available to all patients with ovarian cancer regardless of their genetic profile for treatment and maintenance therapy. With this expanded information about germline mutations, somatic mutations and LOH status may serve a new role, no longer as an indicator of who is eligible for treatment with this class of drugs but instead as a biomarker for treatment efficacy. This information would allow individualized patient counseling where the provider can offer information about the magnitude of benefit given the patient’s genetic profile in the context of risk of side effects.

In summary both somatic and germline testing offer clinically important and not entirely overlapping information. One strategy is to start with somatic tumor testing and use this to triage additional testing. Those patients found to have HRD gene mutations on somatic testing can be referred to genetics to establish whether the mutation is somatic or germline. This strategy would allow for rapid access to genomic information that can guide targeted treatment decisions especially with the recent approval of rucaparib for BRCA mutation carriers, and reduce the burden on genetic counselors who would then only see patients with positive somatic testing. Whether this proposed strategy is feasible based on availability of testing resources and cost is unknown and must be examined in future studies. Furthermore, given recent niraparib data this strategy may not be needed as therapeutic benefit was noted in the maintenance setting in all patients with ovarian cancer regardless of BRCA or HRD status.

### Future directions, can somatic testing replace germline testing?

Currently, as stated above, somatic tumor testing can provide information only about genetic aberrations in the tumor, however as NGS continues to become increasing widespread its applications will likely broaden. Stadler et al. [67] recently published their experience with tumor genomic profiling via NGS panels in 224 patients with colorectal cancer. Previously in colorectal cancer NGS panels were used to identify *KRAS*, *NRAS* and *BRAF* somatic mutations and parallel MMR protein assessment via ether immunohistochemistry (IHC) or microsatellite instability (MSI) analysis was used for initial screening of Lynch syndrome. Mismatch repair deficiency (MMR-D) occurs in 15–20% of colorectal tumors with one-quarter resulting from Lynch syndrome [68]. Stadley and colleagues discovered that by utilizing an expanded NGS panel with 341-genes, a specific mutational load cutoff could reliably identify tumors with DNA MMR-D. This was not surprising given that a deficiency in the DNA MMR apparatus would be expected to result in more uncorrected mismatched bases and thus a greater rate of mutations. The authors concluded that the use of multigene tumor panels may obviate the need for parallel MMR protein assessment and thus make the molecular work-up patients with colorectal cancer more efficient and cost-effective. Identifying MMR-D is becoming particularly important as it may serve as a biomarker for response to immunotherapy with checkpoint inhibitors in colorectal and endometrial cancer [69, 70].

If somatic tumor profiling can replace MMR assessment, the next question is whether or not somatic tumor profiling can replace germline testing altogether. Currently none of the tumor profiling platforms offer information on germline mutational status however this may change. The feasibility of this question was evaluated with a study comparing deep, uniform NGS sequencing of tumor tissue compared to Clinical Laboratory Improvement Amendments (CLIA)-certified, NGS-based germline exon sequencing of 236-cancer related genes. A computational method could predict the somatic status of mutations from tumor tissue without the need for a matched normal control with an accuracy of greater than 95%. As tumor profiling for this indication is permitted under investigational use only and is not CLIA-certified, the authors concluded that NGS tumor sequencing can indicate which patients require additional work-up for germline testing [71]. However, with further analysis and certification, clinicians will likely be able to use somatic testing to definitively predict germline status. While this approach to testing may not make sense for cancers with a low prevalence of hereditary causation, it is very applicable and exciting in ovarian cancer where up to a quarter of patients carry a germline mutation. The FDA accelerated approvals of olaparib and rucaparib came with specified companion diagnostic tests however there are multiple available next-generation sequencing assays and algorithms for defining LOH. Whether or not approval for specific targeted therapies will mandate a specific companion test and how physicians choose the most appropriate diagnostic test remains unclear. Methods to combine assays and validate data across the various PARP inhibitors, so one cost-effective test can be utilized makes logical sense.

Finally, an important remaining area of uncertainty is the role of epigenetic alterations in EOC. HRD can result from genomic processes that are distinct from germline and somatic mutations in HR genes. For example *BRCA1/2* silencing can occur via indirect mechanisms including promoter methylation and interactions with other proteins involved in DNA repair. The clinical implications of epigenetic changes remain unclear with conflicting data in the literature. Chiang et al. [72] found that *BRCA1* promoter hypermethylation resulted in significantly shorter survival when compared to *BRCA1* germline mutations and *BRCA1* wild-type without promoter hypermethylation. In contrast, TCGA found no survival difference with *BRCA1* hypermethylation [7] and Cunningham et al. [27] observed that *BRCA1* methylated cases had a survival benefit compared to germline wild-type and similar to that of the germline mutation cases. Prospective studies are necessary to better address the clinical significance of epigenetic alterations in the HR pathway.

## Conclusions

It is clear that the knowledge of both germline and somatic mutation status is becoming increasingly important in the management of patients with ovarian cancer. The experience with PARP inhibitors in ovarian cancer clinical trials demonstrates that the targeted therapeutic effect is beyond just BRCA germline mutations and more widely applicable to HRD. Whether epigenetic modifications to HRD genes confer the same therapeutic sensitivity remains unclear however future studies will likely allow for a better understanding of their underlying mechanisms and clinical significance. The list of ovarian cancer-associated genes continues to grow and the literature currently suggests that up to one in four women with ovarian cancer will have a germline HR deficiency and an additional 5–7% will have a somatic HR deficiency. Currently, these patients with recurrent disease are candidates for approved therapy with PARP inhibitors, however in the near future there will likely be a growing list of targeted therapies available for ovarian cancer patients. As a result, infrastructure for widespread somatic HRD testing in routine clinical practice, as is the case for HER2 in breast cancer and epidermal growth factor receptor in lung cancer, should be supported [8]. Furthermore, in the maintenance setting where PARP inhibitors may be useful in all patients as noted the NOVA trial, HRD testing maybe useful for counseling to understand the magnitude of the benefit in context with the risks of side effects. Both germline and somatic tumor testing provide important and non-overlapping clinical information. Further research is necessary to address whether somatic testing can completely replace germline testing or if the practice of universal somatic tumor testing followed by directed germline confirmation should be implemented.

## Abbreviations

ACMG: American College of Medical Genetics and Genomics
ASCO: American Society of Clinical Oncology
CLIA: Clinical Laboratory Improvement Amendments
EOC: Epithelial ovarian cancer
HRD: Homologous recombination deficiency
IHC: Immunohistochemistry
LOH: Loss of heterozygosity
LST: Large scale transitions
MMR: Mismatch repair
MMR-D: Mismatch repair deficiency
MSI: Microsatellite instability
NCCN: National Comprehensive Cancer Network
NGS: Next generation sequencing
NHEJ: Non-homologous end-joining
NSGC: National Society of Genetic Counselors
PARP: Poly(ADP-ribose)polymerase
SGO: Society of Gynecologic Oncology
TCGA: The Cancer Genome Atlas
VUS: Variants of uncertain significance

## References

[1] American Cancer Society: Cancer Statistics Center. 2016. p. https://cancerstatisticscenter.cancer.org/.

[2] Covens A, Carey M, Bryson P, Verma S, Fung Kee Fung M, Johnston M (2002). Systematic review of first-line chemotherapy for newly diagnosed postoperative patients with stage II, III, or IV epithelial ovarian cancer. Gynecol Oncol.

[3] Gadducci A, Sartori E, Maggino T, Zola P, Landoni F, Fanucchi A (1998). Analysis of failures after negative second-look in patients with advanced ovarian cancer: an Italian multicenter study. Gynecol Oncol.

[4] Audeh MW, Carmichael J, Penson RT, Friedlander M, Powell B, Bell-McGuinn KM (2010). Oral poly(ADP-ribose) polymerase inhibitor olaparib in patients with BRCA1 or BRCA2 mutations and recurrent ovarian cancer: a proof-of-concept trial. Lancet.

[5] Ledermann J, Harter P, Gourley C, Friedlander M, Vergote I, Rustin G (2014). Olaparib maintenance therapy in patients with platinum-sensitive relapsed serous ovarian cancer: a preplanned retrospective analysis of outcomes by BRCA status in a randomised phase 2 trial. Lancet Oncol.

[6] Fong PC, Yap TA, Boss DS, Carden CP, Mergui-Roelvink M, Gourley C (2010). Poly(ADP)-ribose polymerase inhibition: frequent durable responses in BRCA carrier ovarian cancer correlating with platinum-free interval. J Clin Oncol.

[7] Cancer Genome Atlas Research N (2011). Integrated genomic analyses of ovarian carcinoma. Nature.

[8] Moschetta M, George A, Kaye SB, Banerjee S. BRCA somatic mutations and epigenetic BRCA modifications in serous ovarian cancer. Ann Oncol. 2016;27:1449–55. 10.1093/annonc/mdw142 27037296

[9] Lupo B, Trusolino L (1846). Inhibition of poly(ADP-ribosyl)ation in cancer: old and new paradigms revisited. Biochim Biophys Acta.

[10] Wang M, Wu W, Wu W, Rosidi B, Zhang L, Wang H (2006). PARP-1 and Ku compete for repair of DNA double strand breaks by distinct NHEJ pathways. Nucleic Acids Res.

[11] Hoeijmakers JH (2001). Genome maintenance mechanisms for preventing cancer. Nature.

[12] Pennington KP, Swisher EM (2012). Hereditary ovarian cancer: beyond the usual suspects. Gynecol Oncol.

[13] Norquist BM, Harrell MI, Brady MF, Walsh T, Lee MK, Gulsuner S, et al. Inherited mutations in women with ovarian carcinoma. JAMA Oncol. 2015:1–9. 10.1001/jamaoncol.2015.5495 PMC4845939 26720728

[14] Randall LM, Pothuri B (2016). The genetic prediction of risk for gynecologic cancers. Gynecol Oncol.

[15] Hall JM, Lee MK, Newman B, Morrow JE, Anderson LA, Huey B (1990). Linkage of early-onset familial breast cancer to chromosome 17q21. Science.

[16] Alsop K, Fereday S, Meldrum C, DeFazio A, Emmanuel C, George J (2012). BRCA mutation frequency and patterns of treatment response in BRCA mutation-positive women with ovarian cancer: a report from the Australian Ovarian Cancer Study Group. J Clin Oncol.

[17] Gourley C, Michie CO, Roxburgh P, Yap TA, Harden S, Paul J (2010). Increased incidence of visceral metastases in scottish patients with BRCA1/2-defective ovarian cancer: an extension of the ovarian BRCAness phenotype. J Clin Oncol.

[18] Tan DS, Yap TA, Hutka M, Roxburgh P, Ang J, Banerjee S (2013). Implications of BRCA1 and BRCA2 mutations for the efficacy of paclitaxel monotherapy in advanced ovarian cancer. Eur J Cancer.

[19] Risch HA, McLaughlin JR, Cole DE, Rosen B, Bradley L, Fan I (2006). Population BRCA1 and BRCA2 mutation frequencies and cancer penetrances: a kin-cohort study in Ontario, Canada. J Natl Cancer Inst.

[20] Risch HA, McLaughlin JR, Cole DE, Rosen B, Bradley L, Kwan E (2001). Prevalence and penetrance of germline BRCA1 and BRCA2 mutations in a population series of 649 women with ovarian cancer. Am J Hum Genet.

[21] Pal T, Permuth-Wey J, Betts JA, Krischer JP, Fiorica J, Arango H (2005). BRCA1 and BRCA2 mutations account for a large proportion of ovarian carcinoma cases. Cancer.

[22] Walsh CS. Two decades beyond BRCA1/2: Homologous recombination, hereditary cancer risk and a target for ovarian cancer therapy. Gynecol Oncol. 2015;137:343–50. 10.1016/j.ygyno.2015.02.017 25725131

[23] Walsh T, Casadei S, Lee MK, Pennil CC, Nord AS, Thornton AM (2011). Mutations in 12 genes for inherited ovarian, fallopian tube, and peritoneal carcinoma identified by massively parallel sequencing. Proc Natl Acad Sci U S A.

[24] Song H, Dicks E, Ramus SJ, Tyrer JP, Intermaggio MP, Hayward J (2015). Contribution of Germline Mutations in the RAD51B, RAD51C, and RAD51D Genes to Ovarian Cancer in the Population. J Clin Oncol.

[25] Hennessy BT, Timms KM, Carey MS, Gutin A, Meyer LA, Flake DD (2010). Somatic mutations in BRCA1 and BRCA2 could expand the number of patients that benefit from poly (ADP ribose) polymerase inhibitors in ovarian cancer. J Clin Oncol.

[26] Pennington KP, Walsh T, Harrell MI, Lee MK, Pennil CC, Rendi MH (2014). Germline and somatic mutations in homologous recombination genes predict platinum response and survival in ovarian, fallopian tube, and peritoneal carcinomas. Clin Cancer Res.

[27] Cunningham JM, Cicek MS, Larson NB, Davila J, Wang C, Larson MC (2014). Clinical characteristics of ovarian cancer classified by BRCA1, BRCA2, and RAD51C status. Sci Rep.

[28] Hampel H, Bennett RL, Buchanan A, Pearlman R, Wiesner GL (2015). Guideline Development Group ACoMG, et al. A practice guideline from the American College of Medical Genetics and Genomics and the National Society of Genetic Counselors: referral indications for cancer predisposition assessment. Genet Med.

[29] Lu KH, Wood ME, Daniels M, Burke C, Ford J, Kauff ND (2014). American Society of Clinical Oncology Expert Statement: collection and use of a cancer family history for oncology providers. J Clin Oncol.

[30] Daly MB, Pilarski R, Axilbund JE, Berry M, Buys SS, Crawford B (2016). Genetic/familial high-risk assessment: breast and ovarian, version 2.2015. J Natl Compr Canc Netw.

[31] Lancaster JM, Powell CB, Chen LM, Richardson DL, Committee SGOCP (2015). Society of Gynecologic Oncology statement on risk assessment for inherited gynecologic cancer predispositions. Gynecol Oncol.

[32] Blackburn HL, Schroeder B, Turner C, Shriver CD, Ellsworth DL, Ellsworth RE (2015). Management of incidental findings in the era of next-generation sequencing. Curr Genomics.

[33] Norquist BM, Swisher EM (2015). More genes, more problems? Benefits and risks of multiplex genetic testing. Gynecol Oncol.

[34] Collins VR, Meiser B, Ukoumunne OC, Gaff C, St John DJ, Halliday JL (2007). The impact of predictive genetic testing for hereditary nonpolyposis colorectal cancer: three years after testing. Genet Med.

[35] Watson M, Kash KM, Homewood J, Ebbs S, Murday V, Eeles R (2005). Does genetic counseling have any impact on management of breast cancer risk?. Genet Test.

[36] Pal T, Lee JH, Besharat A, Thompson Z, Monteiro AN, Phelan C (2014). Modes of delivery of genetic testing services and the uptake of cancer risk management strategies in BRCA1 and BRCA2 carriers. Clin Genet.

[37] Hadley DW, Jenkins JF, Dimond E, de Carvalho M, Kirsch I, Palmer CG (2004). Colon cancer screening practices after genetic counseling and testing for hereditary nonpolyposis colorectal cancer. J Clin Oncol.

[38] Schwartz MD, Lerman C, Brogan B, Peshkin BN, Halbert CH, DeMarco T (2004). Impact of BRCA1/BRCA2 counseling and testing on newly diagnosed breast cancer patients. J Clin Oncol.

[39] Calzone KA, Prindiville SA, Jourkiv O, Jenkins J, DeCarvalho M, Wallerstedt DB (2005). Randomized comparison of group versus individual genetic education and counseling for familial breast and/or ovarian cancer. J Clin Oncol.

[40] Armstrong J, Toscano M, Kotchko N, Friedman S, Schwartz MD, Virgo KS (2015). Utilization and outcomes of BRCA genetic testing and counseling in a national commercially insured population: the ABOUT study. JAMA Oncol.

[41] Hilgart JS, Coles B, Iredale R. Cancer genetic risk assessment for individuals at risk of familial breast cancer. Cochrane Database Syst Rev. 2012;15. 10.1002/14651858.CD003721.pub3 PMC7154385 22336791

[42] Braithwaite D, Emery J, Walter F, Prevost AT, Sutton S (2004). Psychological impact of genetic counseling for familial cancer: a systematic review and meta-analysis. J Natl Cancer Inst.

[43] DeMarco TA, Peshkin BN, Mars BD, Tercyak KP (2004). Patient satisfaction with cancer genetic counseling: a psychometric analysis of the Genetic Counseling Satisfaction Scale. J Genet Couns.

[44] Miller CE, Krautscheid P, Baldwin EE, Tvrdik T, Openshaw AS, Hart K (2014). Genetic counselor review of genetic test orders in a reference laboratory reduces unnecessary testing. Am J Med Genet A.

[45] Cragun D, Camperlengo L, Robinson E, Caldwell M, Kim J, Phelan C (2015). Differences in BRCA counseling and testing practices based on ordering provider type. Genet Med.

[46] Weitzel JN, McCaffrey SM, Nedelcu R, MacDonald DJ, Blazer KR, Cullinane CA (2003). Effect of genetic cancer risk assessment on surgical decisions at breast cancer diagnosis. Arch Surg.

[47] Lynparza prescribing information. Lynparza [package insert]. Wilmington, DE: AstraZeneca Pharmaceuticals LP. 2014. Available at http://www.azpicentral.com/Lynparza/pi_lynparza.pdf#page=1.

[48] Mirza MR, Monk BJ, Herrstedt J, Oza AM, Mahner S, Redondo A, et al. Niraparib maintenance therapy in platinum-sensitive, recurrent ovarian cancer. N Engl J Med. 2016;375:2154-2164. 10.1056/NEJMoa1611310 27717299

[49] Vasen HF, Abdirahman M, Brohet R, Langers AM, Kleibeuker JH, van Kouwen M (2010). One to 2-year surveillance intervals reduce risk of colorectal cancer in families with Lynch syndrome. Gastroenterology.

[50] Schmeler KM, Lynch HT, Chen LM, Munsell MF, Soliman PT, Clark MB (2006). Prophylactic surgery to reduce the risk of gynecologic cancers in the Lynch syndrome. N Engl J Med.

[51] Engel C, Rahner N, Schulmann K, Holinski-Feder E, Goecke TO, Schackert HK (2010). Efficacy of annual colonoscopic surveillance in individuals with hereditary nonpolyposis colorectal cancer. Clin Gastroenterol Hepatol.

[52] Abkevich V, Timms KM, Hennessy BT, Potter J, Carey MS, Meyer LA (2012). Patterns of genomic loss of heterozygosity predict homologous recombination repair defects in epithelial ovarian cancer. Br J Cancer.

[53] Birkbak NJ, Wang ZC, Kim JY, Eklund AC, Li Q, Tian R (2012). Telomeric allelic imbalance indicates defective DNA repair and sensitivity to DNA-damaging agents. Cancer Discov.

[54] Popova T, Manie E, Rieunier G, Caux-Moncoutier V, Tirapo C, Dubois T (2012). Ploidy and large-scale genomic instability consistently identify basal-like breast carcinomas with BRCA1/2 inactivation. Cancer Res.

[55] Schwarz RF, Ng CK, Cooke SL, Newman S, Temple J, Piskorz AM (2015). Spatial and temporal heterogeneity in high-grade serous ovarian cancer: a phylogenetic analysis. PLoS Med.

[56] Lin K, Sun J, Maloney L, Goble S, Oza A, Coleman R, Scott C, Robillard L, Mann E, Isaacson J, Harding T (2015). 2710 Quantification of genomic loss of heterozygosity enables prospective selection of ovarian cancer patients who may derive benefit from the PARP inhibitor rucaparib. Eur J Cancer.

[57] Gelmon KA, Tischkowitz M, Mackay H, Swenerton K, Robidoux A, Tonkin K (2011). Olaparib in patients with recurrent high-grade serous or poorly differentiated ovarian carcinoma or triple-negative breast cancer: a phase 2, multicentre, open-label, non-randomised study. Lancet Oncol.

[58] Kummar S, Oza AM, Fleming GF, Sullivan DM, Gandara DR, Naughton MJ (2015). Randomized Trial of Oral Cyclophosphamide and Veliparib in High-Grade Serous Ovarian, Primary Peritoneal, or Fallopian Tube Cancers, or BRCA-Mutant Ovarian Cancer. Clin Cancer Res..

[59] Swisher EM, Lin KK, Oza AM, Scott CL, Giordano H, Sun J (2017). Rucaparib in relapsed, platinum-sensitive high-grade ovarian carcinoma (ARIEL2 Part 1): an international, multicentre, open-label, phase 2 trial. Lancet Oncol.

[60] Ledermann J, Harter P, Gourley C, Friedlander M, Vergote I, Rustin G (2012). Olaparib maintenance therapy in platinum-sensitive relapsed ovarian cancer. N Engl J Med.

[61] Collins FS, Varmus H (2015). A new initiative on precision medicine. N Engl J Med.

[62] Borzekowski DL, Guan Y, Smith KC, Erby LH, Roter DL (2014). The Angelina effect: immediate reach, grasp, and impact of going public. Genetics Med.

[63] Meyer LA, Anderson ME, Lacour RA, Suri A, Daniels MS, Urbauer DL (2010). Evaluating women with ovarian cancer for BRCA1 and BRCA2 mutations: missed opportunities. Obstet Gynecol.

[64] Metcalfe KA, Fan I, McLaughlin J, Risch HA, Rosen B, Murphy J (2009). Uptake of clinical genetic testing for ovarian cancer in Ontario: a population-based study. Gynecol Oncol.

[65] Buchanan AH, Rahm AK, Williams JL (2016). Alternate service delivery models in cancer genetic counseling: a mini-review. Front Oncol.

[66] Watson MU CH, Tillmanns T, Reed ME, Smiley L, Covington R (2016). The implementation of video-assisted genetic counseling for ovarian, fallopian, and peritoneal cancer patients Society of Gynecologic Oncology Annual Meeting on Women’s Cancer San Diego.

[67] Stadler ZK, Battaglin F, Middha S, Hechtman JF, Tran C, Cercek A (2016). Reliable detection of mismatch repair deficiency in colorectal cancers using mutational load in next-generation sequencing panels. J Clin Oncol.

[68] Hampel H, Frankel WL, Martin E, Arnold M, Khanduja K, Kuebler P (2005). Screening for the Lynch syndrome (hereditary nonpolyposis colorectal cancer). N Engl J Med.

[69] Le DT, Uram JN, Wang H, Bartlett BR, Kemberling H, Eyring AD (2015). PD-1 blockade in tumors with mismatch-repair deficiency. N Engl J Med.

[70] A.N. Fader LAD, D.K. Armstrong, E.J. Tanner III, J. Uram, A. Eyring, H. Wang, G. Fisher, T. Greten and D. Le. Preliminary results of a phase II study: PD-1 blockade in mismatch repair–deficient, recurrent or persistent endometrial cancer. Society of Gynecologic Oncology Annual Meeting on Women’s Cancer: Late-breaking abstract sessions 3; 2016.

[71] James X. Sun GF, Kai Wang, Jeffrey S. Ross, Vincent A. Miller, Philip J. Stephens, Doron Lipson, Roman Yelensky. Abstract 1893: A computational method for somatic versus germline variant status determination from targeted next-generation sequencing of clinical cancer specimens without a matched normal control. Proceedings of the 105th Annual Meeting of the American Association for Cancer Research; 2014 Apr 5–9. San Diego, CA 2014.

[72] Chiang JW, Karlan BY, Cass L, Baldwin RL (2006). BRCA1 promoter methylation predicts adverse ovarian cancer prognosis. Gynecol Oncol.

[73] Kaye SB, Lubinski J, Matulonis U, Ang JE, Gourley C, Karlan BY, et al. Phase II, open-label, randomized, multicenter studycomparing the efficacy and safety of olaparib, a poly (ADP-ribose) polymerase inhibitor, and pegylated liposomal doxorubicin in patients with BRCA1 or BRCA2mutations and recurrent ovarian cancer. J Clin Oncol. 2012;30:372–9. 10.1200/JCO.2011.36.9215 22203755

[74] Oza AM, Cibula D, Benzaquen AO, Poole C, Mathijssen RH, Sonke GS, et al. Olaparib combined with chemotherapy for recurrent platinum-sensitive ovarian cancer: a randomised phase 2 trial. Lancet Oncol. 2015;16:87–97. 10.1016/S1470-2045(14)71135-0 25481791

[75] Coleman RL, Sill MW, Bell-McGuinn K, Aghajanian C, Gray HJ, Tewari KS, et al. A phase II evaluation of the potent, highly selective PARP inhibitor veliparib in the treatment of persistent or recurrent epithelial ovarian, fallopian tube, or primary peritoneal cancer in patients who carry a germline BRCA1 or BRCA2 mutation - An NRG Oncology/Gynecologic Oncology Group study. Gynecol Oncol. 2015;137:386–91. 10.1016/j.ygyno.2015.03.042 PMC4447525 25818403

[76] Kaufman B, Shapira-Frommer R, Schmutzler RK, Audeh MW, Friedlander M, Balmaña J, et al. Olaparib monotherapy in patients with advanced cancer and a germline BRCA1/2 mutation. J Clin Oncol. 2015; 33:244–50. 10.1200/JCO.2014.56.2728 PMC6057749 25366685

